# Salvage circumferential endoscopic submucosal dissection for recurrent, strictured Barrett’s neoplasia

**DOI:** 10.1055/a-2916-9976

**Published:** 2026-08-04

**Authors:** Sandra Nagl, Helmut Messmann

**Affiliations:** 1 Internal Medicine III – Gastroenterology 39694 University Hospital AugsburgAugsburgBYGermany

A 76-year-old female patient with a history of heartburn underwent upper endoscopy for ongoing vomiting and was first diagnosed with Barrett’s esophagus C14M14. Random biopsies revealed high-grade dysplasia (HGD). She subsequently underwent endoscopic mucosal resection (EMR). Histology showed adenocarcinomas (T1a, L0, V0, G1, and R0). Follow-up surveillance endoscopy demonstrated recurrent/residual HGD, leading to an additional piecemeal-EMR. As a result, the patient developed a mid-esophageal, endoscopically non-traversable stricture. A balloon dilatation was performed up to a diameter of 12 mm. During surveillance endoscopy, early adenocarcinoma was detected within and below the stenotic segment by targeted biopsy. The patient was then referred to our center for further management. Here, C14M14 Barrett’s esophagus with a mid-esophageal stricture of 2 cm length was described. Endoscopic passage was only possible after further dilatation of the stricture. Biopsies confirmed adenocarcinoma within the stricture and distal to it. In addition, multifocal visible lesions above the stricture were observed. Staging with a computed tomographic scan showed no pathological lymph nodes. The accurate assessment of tumor infiltration by endoscopic ultrasound was not feasible due to the stricture. After multidisciplinary discussion regarding treatment options, risks, and benefits, a circumferential endoscopic submucosal dissection (ESD) with concomitant stricture resection was performed, removing the entire Barrett’s segment except for 2 cm of distal, non-suspicious Barrett’s mucosa. ESD was completed without complications, and the fibrotic circumferential segment, including the mid-esophageal, non-traversable stricture, was removed enbloc ( Video 1 ). Local triamcinolone was injected into the resection bed. Histopathology revealed the adenocarcinoma pT1a (m3), pNx, L0, V0, Pn0, G1, and R0. The patient was discharged 48 hours post-ESD on a liquid/soft diet with a tapered oral prednisolone regimen. Follow-up endoscopies at 2, 4, 8 and 12 weeks demonstrated a healing resection ulcer with complete stricture resolution and no evidence of re-stenosis. The patient reported no further dysphagia.

**Video 1** Circumferential ESD with en bloc resection of a fibrotic mid-esophageal stricture and Barrett’s-associated early adenocarcinoma, followed by complete stricture resolution without re-stenosis. Video URI: .


Endoscopy_UCTN_Code_TTT_1AO_2AG_3AD

